# Four millennia of dairy surplus and deposition revealed through compound-specific stable isotope analysis and radiocarbon dating of Irish bog butters 

**DOI:** 10.1038/s41598-019-40975-y

**Published:** 2019-03-14

**Authors:** Jessica Smyth, Robert Berstan, Emmanuelle Casanova, Finbar McCormick, Isabella Mulhall, Maeve Sikora, Chris Synnott, Richard P. Evershed

**Affiliations:** 1 School of Archaeology, University College Dublin, Newman Building, Belfield, Dublin 4, Ireland; 2 Organic Geochemistry Unit, School of Chemistry, University of Bristol, Cantock’s Close, Bristol, BS8 1TS UK; 3 Present Address: Elementar UK, Isoprime House, Earl Road, Cheadle Hulme, SK8 6PT UK; 4 Archaeology and Palaeoecology, School of Natural and Built Environment, Queen’s University, Belfast, BT7 1NN UK; 5 National Museum of Ireland, Kildare Street, Dublin 2, Ireland; 6 Emeritus Professor, Process and Chemical Engineering Department, University College Cork, Western Road, Cork, Ireland

## Abstract

Bog butters are large white or yellow waxy deposits regularly discovered within the peat bogs of Ireland and Scotland. They represent an extraordinary survival of prehistoric and later agricultural products, comprising the largest deposits of fat found anywhere in nature. Often found in wooden containers or wrapped in animal bladders, they are considered to have been buried intentionally by past farming communities. While previous analysis has determined that Irish bog butters derive from animal fat, their precise characterisation could not be achieved due to diagenetic compositional alterations during burial. Via compound-specific stable isotope analysis, we provide the first conclusive evidence of a dairy fat origin for the Irish bog butter tradition, which differs from bog butter traditions observed elsewhere. Our research also reveals a remarkably long-lived tradition of deposition and possible curation spanning at least 3500 years, from the Early Bronze Age (c. 1700 BC) to the 17^th^ century AD. This is conclusively established via an extensive suite of both bulk and compound-specific radiocarbon dates.

## Introduction

**Figure 1:**
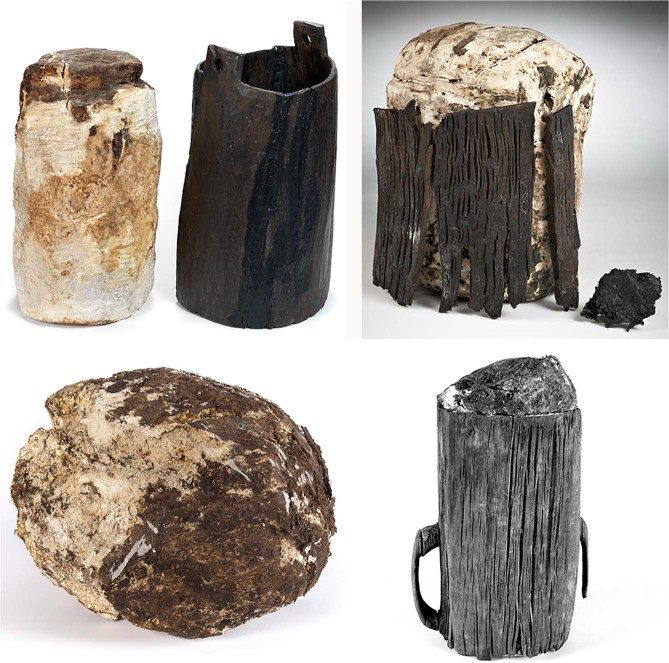
Examples of bog butter recovered from Irish contexts. Clockwise from top left: Rosberry, Co. Kildare (IB18), dated to 360–200 BC and deposited in a keg; Muckanagh, Co. Mayo (IB6), dated to AD 775–895 and associated with a wooden container; Tumgesh, Co. Mayo, deposited in a wooden mether; Shannagurraun, Co. Galway (IB8), dated to AD 960–1040 and wrapped in animal bladder. All dates this study. Images provided by kind permission of the National Museum of Ireland.

Bog butters are large, white to yellow waxy deposits regularly recovered from the peat bogs of Ireland and Scotland, often found in wooden containers or wrapped in bark or animal membranes (Fig. 1). With recorded weights of up to 23 kg (and several examples that may be larger), bog butters were first documented in the 17^th^ century; the total number recovered to date may approach 500 specimens^1,2^. Published radiocarbon determinations on Irish bog butters show activity spanning the Iron Age to the post-medieval period^3,4^ with folk accounts indicating survival into the 19th century^5,6^. While the reasons behind their deposition continue to be debated^1,2^, the remarkable preservative properties of peat bogs are well known^7^ and several post-medieval accounts mention the practice of storing butter in bogs to be consumed at a later date, whether by necessity or as a delicacy^8–10^. Early medieval Irish law tracts list butter as one of the products payable as food rents^11^, which may have needed to be stockpiled or stored. Parallels have also been drawn with the widespread deposition of metal and other objects in wetlands during the Bronze Age and Iron Age, often assumed to be votive or ritual acts^5,12–14^.

More than a century of chemical analyses has successfully determined that bog butters are derived from animal fat, although until recently the precise origins of bog butters could not be established due to diagenetic alterations during burial. The conclusion of early attempts was that they resembled adipocere rather than butter^15–19^. Like bog butter, the chemical composition of adipocere is dominated by saturated fatty acids (mainly palmitic acid; C_16:0_ and stearic acid; C_18:0_), with smaller amounts of unsaturated fatty acids (mainly oleic acid; *Z*-C_18:1_), hydroxy fatty acids (mainly 10-hydroxystearic acid; C_18:0_-OH) and intact triacylglycerols^19–22^. In 2004, chemical analysis of nine Scottish bog butters using compound-specific stable carbon isotope measurements demonstrated six of the bog butters derived from a ruminant dairy source and three from ruminant carcass fat (tallow)^23^. Here we report on analyses undertaken on 32 Irish bog butters (Table 1), with an accompanying programme of radiocarbon dating, to ascertain if similar practices took place in Ireland and if trends through time could be observed.

**Table 1:** Irish bog butters sampled for this study.

Code	County	Townland	Museum no.	Associated container
IB1	Offaly	Esker More	1998:62	
IB2	Offaly	Knockdrin	1998:63	Bark adhering to surface
IB3	Offaly	Ballindown	1986:125	
IB4	Laois	Baunaghra	1986:40	
IB5	Laois	Colt	1986:58	
IB6	Mayo	Muckinagh	2013:148.1-0.2	Wooden container?
IB7	Galway	Drinaun	1983:29.1-0.2	Bladder?
IB8	Galway	Shannagurraun	1983:28	Bladder
IB9	Kildare	Newtownbert	1967:102–103	Wicker basket
IB10	Mayo	Tawnagh Beg	1940:44	Mether
IB11	Mayo	Mullagh	1929:1343	Wooden
IB12	Roscommon	Rosmoylan	1962:101	Keg
IB13	Monaghan	Corlea	1965:275	Keg
IB14	Kerry	Tullamore	1954:16.1-0.2	Tub
IB15	Kildare	Hawkfield	1986:36	
IB16	Galway	Killeenan More	1939:994	Bowl
IB17	Mayo	Sheskin	1958:11	Bladder with bark
IB18	Kildare	Rosberry	1970:32	Keg with cord
IB19	Galway	Teernakill Bog	1925:14	Plunge churn
IB20	Donegal	Ards Beg	1987:112	Tub
IB21	Mayo	Ballyguin	1943:314–5	Two bladders in wooden container
IB22	Tipperary	Derrycoogh	1991:13	Bark wrappings
IB23	Mayo	Derryloughan	M1948:4	Wooden
IB24	Kildare	Killinagh	1929:1298	Wooden
IB25	Limerick	Glennacowan	1943:54	
IB26	Meath	Ardanew	1930:195	Keg
IB27	Mayo	Rosdoagh	1968:440 A	Stave tub
IB28	Leitrim		2018:29–30	Bark
IB29	Mayo	Gowlaune	2018:27–28	Bark
IB30	Sligo	Cloncoose	2007:38	Bark
IB31	Mayo	Derragh	2007:39	
IB32	Mayo	Knockmoyle	1986:39	Keg

## Classification of Degraded Animal Fat Remains Using Stable Isotopes

**Figure 2:**
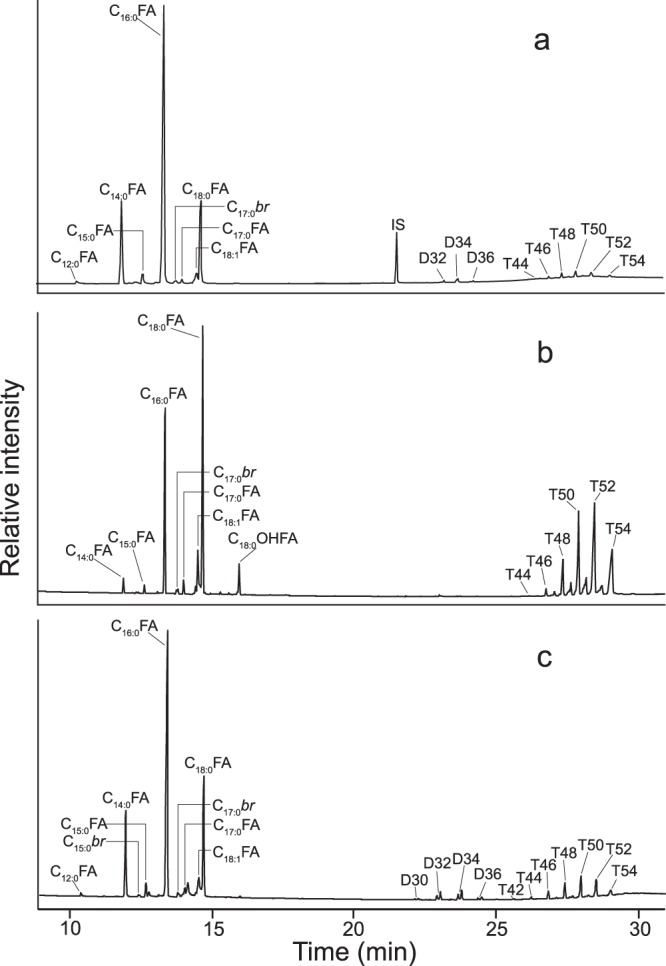
Partial high-temperature gas chromatograms of trimethylsilylated extracts from: (**a**) bog butter IB20, and adipoceres produced from (**b**) mutton fat and (**c**) New Zealand butter^19^. Chromatographic peak identities are: C_12:0_ FA to C_18:0_ FA, saturated straight chain fatty acids with 12 to 18 carbons, respectively; C_18:1_ FA, mono-unsaturated fatty acid with 18 carbon atoms; C_15:0br_ and C_17:0br_, branched chain fatty acids with 15 and 17 carbon atoms, respectively; C_18:0_ OHFA, hydroxy fatty acid containing 18 carbon atoms; D_32_ to D_36_, diacylglycerols with 32 to 36 acyl carbons, respectively; T_44_ to T_54_, triacylglycerols with 44 to 54 acyl carbons, respectively; IS, internal standard, *n*-tetratricontane (*n*-C_34_).

In ruminant (e.g. cattle and sheep) and non-ruminant animals (e.g. pigs), adipose tissue is the main site for the storage of lipids, with triacylglycerols being by far the most abundant constituent, making up over 95% of the total lipids present^24^. These triacylglycerols comprise of three fatty acids attached via ester linkages to a glycerol backbone, where the fatty acids mainly consist of an even number of acyl carbon atoms. In animal fats, acyl carbon chain lengths of C_16_ and C_18_ generally dominate^24,25^. In addition to adipose tissue, ruminant milk fats are also predominantly made up of triacylglycerols, but with a higher proportion of short chain fatty acids^26^. The presence of these short chain fatty acids (C_4:0_ to C_12:0_) in ruminant milk fats is in direct contrast to equivalent adipose fats, which contain very few fatty acids with chain lengths less than C_14:0_^26^. Compared to other biochemical classes such as carbohydrates and proteins, the relative hydrophobic nature of lipids ensures their more frequent survival during archaeological timescales, with one of the most common finds being that of degraded animal fats^27^. By using high-temperature gas chromatography (HTGC) and GC/mass spectrometry (GC/MS), such organic remains can easily be classified as deriving from animal fats based on the identification and distribution of free fatty acids (mainly C_16:0_ and C_18:0_) and any remaining acylglycerols. Identification to species or fat type is much more difficult due to diagenetic transformations that occur during burial^28,29^. The heavier of the low molecular weight triacylglycerols, with acyl carbon numbers of C_40_ and C_42_, occasionally survive and can suggest a ruminant dairy fat origin rather than an adipose fat. However, dairy fat residues surviving archaeological timescales are often indistinguishable from adipose fat due to the increased solubility and hence preferential loss of the short chain fatty acids; for each additional methylene group of a fatty acid there is a fourfold decrease in its solubility (Fig. 2)^30^.

With the aid of stable carbon isotope determinations (δ^13^C values) on individual fatty acids, distinctions between the adipose fats of different animals can readily be achieved, first demonstrated by comparing the δ^13^C values of lipid residues extracted from medieval dripping dishes and lamps with modern reference fats from pigs and ruminant animals^31,32^. Since then, traces of ruminant dairy fats (with δ^13^C values ca. 2 to 4‰ less than adipose fat) have been successfully identified in a large number of pottery vessels from throughout Europe and the Near East^28,33–35^.

## Results

### Lipid residue analysis & stable carbon isotope measurements

**Figure 3:**
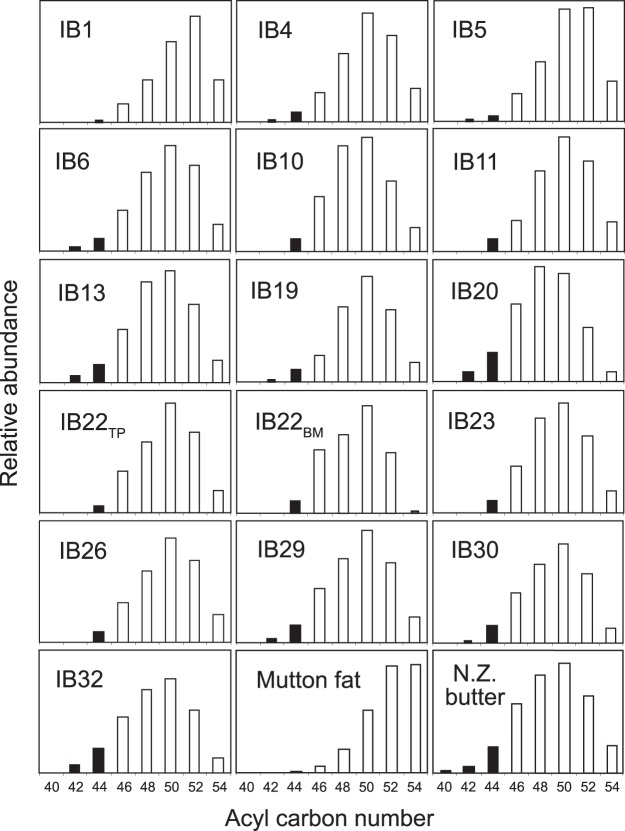
Histograms showing acyl carbon number distributions of triacylglycerols identified in the fifteen Irish bog butters that contained appreciable quantities and, for comparison, from previously reported *in vitro* adipocere formed from butter and mutton fat^19,23^. Shaded areas represent low molecular weight triacylglycerols, potentially deriving from a ruminant dairy fat. The abundance of each triacylglycerol component was calculated by integrating the peak areas in the HTGC profile.

Lipid compositions of the Irish bog butters were determined through GC and GC/MS analysis of each trimethylsilylated lipid extract (Supplementary Table S1). Similar to the findings of their Scottish equivalents^18,23^, free fatty acids with carbon numbers ranging from C_12_ to C_20_ (even over odd predominance) were the principal lipid components, with palmitic (C_16:0_) and stearic (C_18:0_) acids predominating (Fig. 2). Hydroxystearic acids (mainly 10-hydroxystearic) were also found to be present in seventeen of the bog butter samples with abundances ranging from 0.1% to 10.4% (mean 1.4%) of the total free fatty acids, respectively. These hydroxy fatty acids are known to be produced during adipocere formation^20^ and were also identified in the Scottish bog butter samples^23^. The other significant lipid components present in some of the Irish bog butters were acylglycerols, which demonstrates that hydrolysis to their component fatty acids had not gone to completion; fifteen of the bog butters contained triacylglycerols and eight contained diacylglycerols (Fig. 3). Nine (IB4-6, 13, 19, 20, 29, 30 and 32) of the bog butters were found to consist of triacylglycerol distributions with acyl carbon numbers ranging from C_42_ to C_54_, while the remaining six (IB1, 10, 11, 22, 23 and 26) ranged from C_44_ to C_54_ acyl carbons. Those with acyl carbon distributions ranging from C_42_ to C_54_ are entirely consistent with a degraded dairy fat origin. However, those with acyl carbons ranging from C_44_ to C_54_ are more consistent with a ruminant adipose fat source, although a dairy origin cannot be discounted due to the presence and abundance of the C_44_ triacylglycerol. A more reliable approach in the identification of the Irish bog butter origins was through the measurement of the stable carbon isotope values (δ^13^C values) of their C_16:0_ and C_18:0_ fatty acids. These values were compared against a global database of modern reference animal fats including animals from the UK raised on a pure C_3_ diet (^28^; Supplementary Table S1).

**Figure 4:**
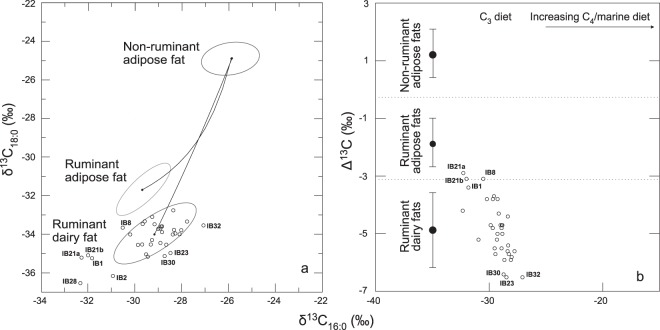
δ^13^C values of methylated individual fatty acids (C_16:0_ and C_18:0_) from sampled Irish bog butters. (**a**) δ^13^C values plotted against reference ellipses (1δ) derived from modern UK animal fats corrected for the contribution of post-industrial carbon by the addition of 1.2‰^58^. (**b**): the same data with Δδ^13^C values (=δ^13^C_18:0_ − δ^13^C_16:0_) plotted against δ^13^C_16:0_ values. Ranges of Δδ^13^C values are based on a global database comprising modern reference animal fats from the UK, Africa, Kazakhstan, Switzerland and the Near East.

The δ^13^C values of the C_16:0_ and C_18:0_ fatty acids from the 32 Irish bog butters were plotted as a scatter graph with confidence ellipses (1δ) representing ranges corresponding to reference non-ruminant adipose fats and ruminant adipose and dairy fats (Fig. 4a). Twenty-four samples plotted within the reference dairy fat ellipse and a further three (IB23, 30, 32) in very close proximity, suggesting a dairy fat origin. The precise origin for bog butter IB8 was unclear as a result of plotting between the reference ellipses of the ruminant adipose and dairy fats. IB1 and IB21 displayed δ^13^C values more similar to a dairy fat origin; however, the δ^13^C values for their C_16:0_ fatty acids were approximately 1‰ more depleted in ^13^C than reference dairy fats. Likewise, samples IB2 and IB28 also revealed values more similar to a ruminant dairy fat origin, but with both their C_16:0_ and C_18:0_ fatty acids more depleted in ^13^C than reference dairy fat values. These bog butters may indeed have a dairy origin, with the observed values occurring as a result of local isotopic differences in the diets of the ancient and modern reference animals. Such variations are negated by comparing the Δ^13^C values (δ^13^C_18:0_ − δ^13^C_16:0_) of the bog butters with the reference fat values (Fig. 4b), and here 26 of the 32 Irish bog butter samples were found to derive from a ruminant dairy origin with all values plotting within the range corresponding to a ruminant dairy fat. A further three bog butters (IB23, 30 and 32) were also likely to have derived from a dairy source as their Δ^13^C values plotted just below the reference dairy fat range. A similar phenomenon has been noted for Δ^13^C values from Irish Neolithic pot lipids and may be due to local environmental factors^35,36^. The remaining bog butters (IB1, 8 and 21) could not be precisely classified as their Δ^13^C values plotted mid-way between the reference ranges for ruminant adipose and dairy fats. While no container was associated with IB1, both IB8 and IB21 were wrapped in animal bladders and their fatty acid δ^13^C values may have been altered by the lipid content of these wrappings. As with the Scottish bog butters^23^, where multiple samples were analysed from each bog butter mass, the homogeneity of bog butters and the robustness of the stable isotope methodology was confirmed. Analyses of sub-samples taken from the top (IB22tp) and bottom (IB22bm) of the same bog butter mass (IB22), revealed analogous results. Bog butter IB21, comprising two masses (IB21a and IB21b) wrapped separately, also provided very similar values.

### Radiocarbon measurements on bulk bog butter samples

**Figure 5:**
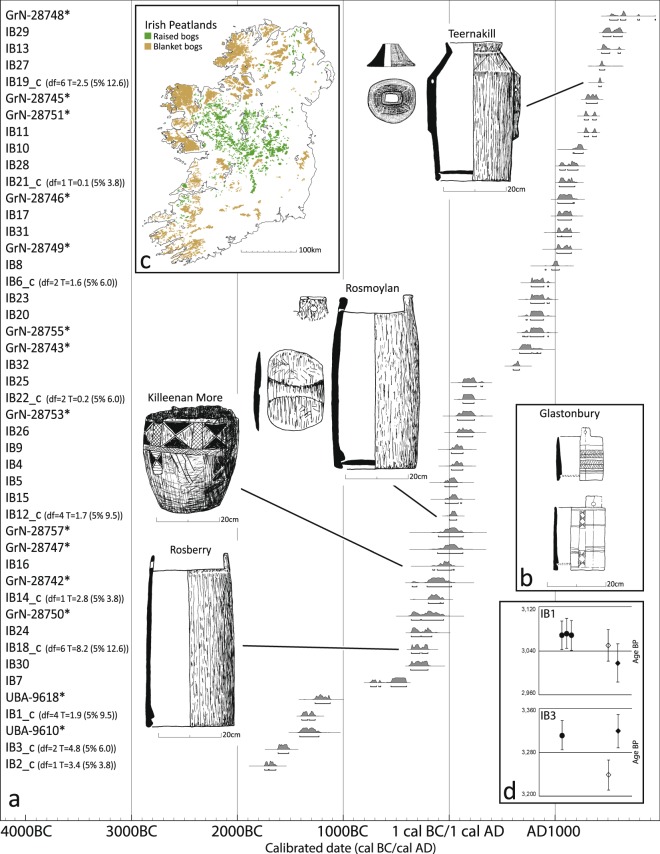
Dating of Irish bog butters. (**a**) Radiocarbon date ranges for Irish bog butters, this study and additional specimens (marked with *; Supplementary Table S2), with associated containers. Date ranges were calibrated using OxCal v4.3.2 and the IntCal13 atmospheric curve^56,57^. ‘C’ denotes calculation of the weighted average of multiple measurements on the same bog butter, with the related chi-squared test results also displayed. T is the chi-squared value calculated and the value given in brackets is the level above which T should not rise in order to be acceptable. Degrees of freedom are given by df (number of dates minus one). All T results are below the respective values for rejection of the contemporaneity hypothesis at 2σ confidence level. (**b**) Iron Age wooden vessels from Glastonbury Lake Village, UK; (**c**) Irish peatland distribution^59^; (**d**) Bulk and compound-specific radiocarbon measurements in years BP for Bronze Age bog butters IB1 and IB3. Dots correspond to bulk dates and diamonds to compound-specific dates. White diamonds = C_16:0_ fatty acids, black diamonds = C_18:0_ fatty acids. Error bars correspond to 1σ analytical uncertainty. Wooden vessels drawn by A. O’Sullivan, map drawn by C. McDermott.

Radiocarbon dating undertaken for this study has provided 50 new measurements on 32 Irish bog butters, adding to the 20 previously published measurements on 19 examples (Supplementary Table S2). Together, they show bog butter manufacture and deposition spanning nearly four thousand years, from 1745–1635 BC (IB2; Knockdrin) to AD 1510–1800 (GrN-28728; Crovehy), generating new insights into both the butters and their associated containers (Fig. 5a). Earwood’s 1997 typology of kegs and churns (originally supported by six radiocarbon dates) holds up extremely well, with the ‘Keg/Tub 2’ type pushed back slightly from the Late to the Middle Iron Age, contemporary with the ‘Keg/Tub 1’ type. Most dramatic is the re-dating of the bowl from Killeenan More, Co. Galway (IB16), presumed to be medieval on the basis of its decoration^2,3^ but shown here to be Middle Iron Age. It joins the straight-sided tubs from Glastonbury Lake Village as rare examples of prehistoric decorated wooden vessels^37,38^ (Fig. 5b). Two of the three bog butters returning Bronze Age dates (IB1, IB3) were measured using both bulk and compound-specific methods (Fig. 5d), conclusively demonstrating that these unusually early dates are not due to contamination or other sample processing issues^39^. However, there is further confirmation of the discrepancy between dates on butter and those on their containers, possibly due to contamination from the polyethylene glycol (PEG) used to consolidate wooden material^3^. Bog butter samples from Rosberry (IB18) and Teernakill (IB19) are both several centuries younger than their containers, although a sample from Rosmoylan (IB12) is a similar age to its associated wooden keg (Supplementary Table S2). While the species (*Alnus sp*.) of the Rosberry keg is noted, none of the previously reported dates on wooden vessels include information on whether heartwood or sapwood was sampled^3,40^.

## Discussion

Our analysis has confirmed that the substances known as ‘bog butter’ in Ireland are indeed butter, which is not as self-evident as one would suppose. While some 17^th^ century sources mention the Irish burying butter in bogs, there are contemporary accounts of Faroe Islanders burying sheep tallow prior to consumption^40^, alongside clear evidence of adipose fat comprising many of the Scottish bog butters. Compound-specific stable isotope analysis provides the only method to conclusively establish Irish bog butter origins. Combining this analysis with radiocarbon measurements, we obtain unparalleled insight into an extremely long-lived activity. Clearly, it is unlikely there was a single reason for the deposition of bog butter over four millennia. Moreover, explanations which seek either a utilitarian or a ritual motivation perpetuate unhelpful categories that may not have applied in the past^41^.

### Bronze age bog butter

Together with two recently dated samples (Supplementary Table S2), this study brings to five the number of Bronze Age bog butters recorded from Ireland. Their date is extremely significant and pushes back depositional activity by as much as 1500 years. Exact locational detail varies, but four of these five bog butters come from Co. Offaly: two are recorded approximately 8 km apart at Ballindown (IB3) and Drinagh townlands, while approximately 45 km to the northeast, two are recorded approximately 12 km apart at Esker More (IB1) and Knockdrin (IB2) townlands. A fifth example was recovered from Clonava townland, Co. Westmeath, approximately 35 km further to the northwest. These very early butter deposits may yet prove to be an isolated phenomenon, although the processing of milk is widespread in Ireland from the Early Neolithic onwards^35,36^ and gradual intensification of dairying over two millennia may have led to substantial surpluses being generated by the Early Bronze Age. The earliest dated sample, Knockdrin (IB2; 1745–1635 BC), and the Drinagh bog butter are both associated with bark, possibly a wrapping or container - a method of storage also evidenced in the Iron Age and Early Medieval period (Supplementary Table S2). In the early 2^nd^ millennium BC, only small, round-based wooden bowls are known and pottery from non-funerary contexts is rare^3,42^, suggesting that deliberate choices were made about the materials used to store surplus food. While the acidic, anaerobic environment of bogs may have been utilised for temporary storage, there are wider patterns of depositional behaviour in the Early Bronze Age to be considered. Strict depositional rules have been observed for gold objects, axes and specialised bladed weapons^43^; foods are an often-ignored category but may have also been infused with symbolism. In this regard, it may be no coincidence that both butter and gold are commonly deposited in bogs (cf^1^).

### Iron age bog butter

Previous work has highlighted an apparent clustering of bog butter deposition in the Iron Age, as well as a possible focus on political and/or natural boundaries^14^. Results from our study bring to 20 the number of recorded Iron Age bog butters (out of 46 dated samples), supporting this first observation although more research is needed to elucidate their relationship to boundaries. More than half (11/20) are associated with vessels, which in Iron Age Britain is recognised as a common category of votive object and argued to be linked to wider symbolic practices around food and agricultural fertility^13,44,45^. It is uncertain if such symbolism permeated the Irish examples, although we note fewer wooden vessels are associated with bog butter in the following Early Medieval period.

### Early medieval and late medieval bog butter

Just under half of the bog butters examined are medieval in date (15 of the 32 samples analysed here; 22 out of 46 total dated samples). Both early and later medieval written sources contain extensive references to dairy products, and butter is often portrayed as a luxury or upper-class food^11^. It is generally included in food-rents, quantities ranging from the fist-size pats of butter listed in 7^th^/8^th^ century law texts to the ‘*yearlie twenty fower methers of butter, and fiftie methers of barlie*’ exacted by Lisgole Abbey, Co. Fermanagh in 1609^11,46^. Interestingly, texts do not mention the practice of depositing butter in bogs, although raiding of butter stores (*imenna*) is periodically recorded; food security must have been an issue for communities, with the storage of butter in bogs perhaps a wise precaution.

In terms of vessels, six of the thirteen Early Medieval (6^th^–12^th^ centuries AD) bog butters were found in wooden containers, the remainder associated with leather, bladder or bark. Written sources indicate bark was commonly used for storing butter, with the Irish word *rúsc* meaning both bark and butter container^11^. The *meadar* or mether, a distinctive quadrangular wooden vessel, appears in the Late Medieval period and is associated with two dated bog butters: Tawnagh Beg (IB10) and Goolamore, both from Co. Mayo (Supplementary Table S2). Recent research has distinguished between a ‘drinking-type’ and a ‘container-type’ mether^47^, the former featuring spout-like or fluted corners with two to four handles positioned high on the vessel, and the latter with no fluted corners, two very large low-set handles, and larger. Tawnagh Beg (and possibly Goolamore) is one of the ‘container-type’ methers and its date of AD 1170–1280 provides the earliest known example of either vessel type.

### Post-medieval/modern bog butter

The date ranges of up to three (out of 46) bog butters span the Irish post-medieval period (AD 1550–1850), with several 17^th^ century accounts written by English observers^9,10,48,49^ providing the first mention of bog butter consumption. Experiments suggest that fresh butter deposited in bog conditions deteriorates relatively quickly and achieves a ‘bog butter’ condition in about two years^19^, with aged or altered butters by no means inedible^50,51^. Although our study has not identified any bog butters more recent than the 18^th^ century, such a practice may have survived into the early 20^th^ century in parts of rural Ireland^5^, alongside the very widely documented folk superstitions and traditions associated with dairying and butter-making^6,52–54^.

## Conclusions

Consistent with previous work, this investigation reveals that all sampled Irish bog butter were animal fats, which during burial had been diagenetically altered to resemble adipocere. GC-C-IRMS analyses of these substances revealed that twenty-six (81%) of the Irish specimens could confidently be assigned a ruminant dairy fat origin, with a further three samples (91% in total) probably deriving from a ruminant dairy fat. Only three samples (9%) could not be identified to origin, with their δ^13^C values plotting between ranges expected for ruminant adipose and dairy fats. Deposition of butter in bogs in Ireland dates from at least the Early Bronze Age, a practice that may reflect intensification of a well-established dairying economy and thus increased likelihood of substantial surpluses of butter, a highly perishable but nutritionally valuable resource. Indeed, it may be that the burial of fats in the ground was much more widespread in antiquity than the archaeological record reflects. The survival of major hoards in bogs is consistent with their remarkable preservative properties; while burial of similar butter deposits in soil may well have achieved a similar goal of preservation in the short term, these deposits would not survive to the present day. The Irish bog butters thus provide a unique encounter with a vitally important agricultural product.

## Materials and Methods

Samples from 32 bog butters (IB1–32) from various locations throughout Ireland were supplied by the National Museum of Ireland, Dublin (Fig. 2). Lipid analysis protocols and instrument conditions were described in detail previously^23^. Briefly, bog butter samples (ca. 1 mg) were extracted in a mixture of chloroform and methanol (2:1 v/v; 10 ml) via ultrasonication and then centrifuged, filtered and evaporated under a gentle stream of N_2_ to yield a total lipid extract (TLE). An aliquot of each TLE was trimethylsilylated using *N,O*-bis(trimethylsilyl) trifluoroacetamide (20 μl; 70°C; 20 min) for analyses via HTGC and GCMS. Further aliquots were saponified using sodium hydroxide in methanol and double distilled water (9:1 v/v; 0.5 M; 2 ml; 70 °C, 1 h) and the fatty acids were converted into fatty acid methyl esters (FAME) using BF_3_/methanol (14% w/v; 100 µl; 70 °C; 1 h) and analysed via GC-C-IRMS to determine their δ^13^C values, which were corrected for added derivative carbon via a mass balance calculation^55^. Bog butters were sampled from the middle of the mass, thus considered free of exogenous contaminant, and ca. 1.2 mg was directly weighed into tin capsules prior to graphitisation to obtain a bulk date. The compound-specific dates on single fatty acids were performed using preparative capillary gas chromatography for the isolation of single compounds^39^. Bog butter samples (bulk and single compounds) were graphitised into an Automated Graphitisation Equipment (AGE3, Ion Plus) and the resulting graphite measured on the BRIS-MICADAS instrument (Ion Plus) at the BRAMS facility in Bristol. Measurements were calibrated using OxCal v4.3.2 and the IntCal13 atmospheric curve^56,57^.

## Supplementary information

Supplementary Tables S1 and S2
