# Supplementary material for: Four millennia of dairy surplus and deposition revealed through compound-specific stable isotope analysis and radiocarbon dating of Irish bog butters
Source: Sci Rep. 2019 Mar 14;9:4559. doi: 10.1038/s41598-019-40975-y (PMC6418298; doi:10.1038/s41598-019-40975-y)
Supplement: Supplementary file 1 — Supplementary Tables S1 and S2 [file 41598_2019_40975_MOESM1_ESM.pdf]

Compound-specific stable isotope analysis and radiocarbon dating of Irish bog butters reveal four millennia of dairy surplus and deposition

J. Smyth, R. Berstan, E. Casanova, F. McCormick, I. Mulhall, M. Sikora, C. Synnott, R.P. Evershed

Supplementary Table S1:
Lipid composition and δ^{13}C values of analysed Irish bog butters. Key to major lipid components: FFA C_x are straight chain free fatty acids of carbon length x, OHFA C_x are hydroxy fatty acids of carbon length x; DAG C_x are diacylglycerols of carbon length x; TAG C_x are triacylglycerols of carbon length x.

Code	Major lipid components	δ$^{13}C_{16:0}$ fatty acid (‰)	δ$^{13}C_{18:0}$ fatty acid (‰)	Assignment
IB1	FFA(C_{12}-C_{20}); OHFA(C_{18}); TAG(C_{44}-C_{54})	-31.84	-35.24	Possible dairy fat
IB2	FFA(C_{12}-C_{18}); OHFA(C_{18}); TAG(C_{44}-C_{54})	-30.95	-36.16	Dairy fat
IB3	FFA(C_{12}-C_{20}); OHFA(C_{18}); TAG(C_{44}-C_{54})	-29.70	-34.53	Dairy fat
IB4	FFA(C_{12}-C_{18}); OHFA(C_{18}); TAG(C_{42}-C_{54})	-28.89	-33.60	Dairy fat
IB5	FFA(C_{12}-C_{18}); OHFA(C_{18}); TAG(C_{42}-C_{54})	-29.17	-33.48	Dairy fat
IB6	FFA(C_{12}-C_{20}); OHFA(C_{18}); DAG(C_{32}-C_{36}); TAG(C_{42}-C_{54})	-29.53	-35.04	Dairy fat
IB7	FFA(C_{12}-C_{18})	-28.92	-33.70	Dairy fat
IB8	FFA(C_{12}-C_{18})	-30.53	-33.66	Possible dairy fat
IB9	FFA(C_{12}-C_{18})	-28.03	-33.79	Dairy fat
IB10	FFA(C_{12}-C_{18}); DAG(C_{32}-C_{36}); TAG(C_{44}-C_{54})	-28.34	-33.77	Dairy fat
IB11	FFA(C_{12}-C_{18}); DAG(C_{32}-C_{36}); TAG(C_{44}-C_{54})	-29.59	-33.33	Dairy fat
IB12	FFA(C_{12}-C_{18})	-29.28	-34.30	Dairy fat
IB13	FFA(C_{14}-C_{20}); DAG(C_{30}-C_{36}); TAG(C_{42}-C_{54})	-29.01	-33.72	Dairy fat
IB14	FFA(C_{14}-C_{18})	-29.88	-34.55	Dairy fat
IB15	FFA(C_{12}-C_{18})	-28.39	-34.01	Dairy fat
IB16	FFA(C_{12}-C_{18})	-28.85	-33.89	Dairy fat
IB17	FFA(C_{12}-C_{18})	-29.27	-33.10	Dairy fat
IB18	FFA(C_{12}-C_{18})	-30.21	-34.01	Dairy fat
IB19	FFA(C_{14}-C_{18}); DAG(C_{30}-C_{36}); TAG(C_{42}-C_{54})	-28.37	-32.76	Dairy fat
IB20	FFA(C_{14}-C_{18}); DAG(C_{32}-C_{36}); TAG(C_{42}-C_{54})	-27.78	-33.34	Dairy fat

IB21a	FFA(C_{12}-C_{18}); OHFA(C_{18})	-32.29	-35.21	Possible dairy fat
IB21b	FFA(C_{12}-C_{18}); OHFA(C_{18})	-32.01	-35.09	Possible dairy fat
IB22tp	FFA(C_{12}-C_{18}); OHFA(C_{18}); TAG(C_{44}-C_{54})	-28.10	-34.00	Dairy fat
IB22bm	FFA(C_{12}-C_{18}); OHFA(C_{18}); TAG(C_{44}-C_{54})	-28.28	-33.96	Dairy fat
IB23	FFA(C_{12}-C_{18}); OHFA(C_{18}); TAG(C_{44}-C_{54})	-28.49	-34.97	Probable dairy fat
IB24	FFA(C_{12}-C_{18}); OHFA(C_{18})	-28.92	-34.45	Dairy fat
IB25	FFA(C_{12}-C_{18}); OHFA(C_{18})	-29.46	-35.16	Dairy fat
IB26	FFA(C_{10}-C_{18}); OHFA(C_{18}); DAG(C_{32}-C_{36}); TAG(C_{44}-C_{54})	-29.33	-34.53	Dairy fat
IB27	FFA(C_{12}-C_{18}); OHFA(C_{18})	-29.68	-33.47	Dairy fat
IB28	FFA(C_{12}-C_{18})	-32.34	-36.53	Dairy fat
IB29	FFA(C_{12}-C_{20}); OHFA(C_{18}); DAG(C_{30}-C_{36}); TAG(C_{42}-C_{54})	-28.67	-34.54	Dairy fat
IB30	FFA(C_{12}-C_{20}); OHFA(C_{18}); TAG(C_{42}-C_{54})	-28.74	-35.13	Probable dairy fat
IB31	FFA(C_{12}-C_{18}); OHFA(C_{18})	-28.85	-33.59	Dairy fat
IB32	FFA(C_{12}-C_{20}); OHFA(C_{18}); TAG(C_{42}-C_{54})	-27.08	-33.54	Probable dairy fat

Supplementary Table S2: Radiocarbon determinations for Irish and Scottish bog butters. All measurements calibrated using OxCal v4.3.2 and IntCal13 atmospheric curve (1, 2)

Code	Townland	County	^{14}C age BP and associated lab code (this study)	Calibrated date range (2σ)	Associated ^{14}C determination (years BP)	Calibrated date range (2σ)	NMI accession no.	Container * Earwood 1997 type	Refs
IB1	Esker More	Offaly	3069±16; BRAMS-1087.1	1405-1275 BC			1998:62		
			3050±30; BRAMS-1087.4.1 ($C_{16:0}$ FA)	1410-1220 BC					
			3018±35; BRAMS-1087.4.2 ($C_{18:0}$ FA)	1400-1120 BC					
IB2	Knockdrin	Offaly	3431±34; BRAMS-1088.1	1880-1840, 1830-1640 BC	3368±22; UBA-9617; butter	1740-1715, 1695-1615 BC	1998:63	Bark adhering to surface	
IB3	Ballindown	Offaly	3308±34; BRAMS-1089.1	1670-1500 BC			1986:125		
			3239±28; BRAMS-1089.2.1 ($C_{16:0}$ FA)	1610-1440 BC					
			3319±31; BRAMS-1089.2.2 ($C_{18:0}$ FA)	1690-1510 BC					
IB4	Baunaghra	Laois	1924±34; BRAMS-1090.1	20 BC-AD 170, AD 200-210			1986:40		3
IB5	Colt	Laois	1962±34; BRAMS-1091.1	50 BC-AD 80, AD 100-130	1880±40; GrN-28752; butter	AD 50-240	1986:58		3-5
IB6	Muckanagh	Mayo	1153±25; BRAMS-1092.1	AD 770-970			2013:148.1-.2	Wooden container	
			1201±29; BRAMS-1092.2.1 ($C_{16:0}$ FA)	AD 719-750, 760-900, 930-940					
			1168±34; BRAMS-1092.2.2 ($C_{18:0}$ FA)	AD 770-970					
IB7	Drinaun	Galway	2422±26; BRAMS-1093.1	750-680, 670-640, 550-400 BC			1983:29.1-.2	Bladder?	3
IB8	Shannagurraun	Galway	1027±27; BRAMS-1153.1	AD 900-920, 960-1040			1983:28	Bladder	3
IB9	Newtownbert	Kildare	1923±27; BRAMS-1154.1	AD 20-140			1967:102-103	Wicker basket	3
IB10	Tawnagh Beg	Mayo	814±25; BRAMS-1094.1	AD 1170-1270			1940:44	Mether	3
IB11	Mullagh	Mayo	670±25; BRAMS-1095.1	AD 1270-1320, 1350-1390			1929:1343	Wooden	
IB12	Rosmoylan	Roscommon	1971±15; BRAMS-1096.1	25-10 BC, 5 BC-AD 75	1940±50; GrA-5458; wood	50 BC-AD 180, 190-220	1962:101	Keg *K2	3, 6-7
			1919±37; BRAMS-1096.2.1 ($C_{16:0}$ FA)	AD 0-180, 190-220					
			1967±40; BRAMS-1096.2.2 ($C_{18:0}$ FA)	50 BC-AD 130					
IB13	Corlea	Monaghan	401±25; BRAMS-1097.1	AD 1430-1520, 1590-1620			1965:275	Keg *K5	3, 8
IB14	Tullamore	Kerry	2087±26; BRAMS-1098.1	180-40 BC	2160±35; GrN-28744; butter	360-90 BC	1954:16.1-.2	Tub *T2	3-5

Code	Townland	County	^{14}C age BP and associated lab code (this study)	Calibrated date range (2σ)	Associated ^{14}C determination (years BP)	Calibrated date range (2σ)	NMI accession no.	Container * Earwood 1997 type	Refs
IB15	Hawkfield	Kildare	1959±26; BRAMS-1099.1	40 BC-AD 90, 100-120			1986:36		3
IB16	Killeenan More	Galway	2035±26; BRAMS-1100.1	160-130 BC, 120 BC-AD 30, 40-50			1939:994	Bowl *FRB	9
IB17	Sheskin	Mayo	945±25; BRAMS-1101.1	AD 1020-1160			1958:11	Bladder with bark	3
IB18	Rosberry	Kildare	2192±15; BRAMS-1102.1	360-270, 260-190 BC	2270±30; GrN-23287; butter<br>2740±50; GrA-5457; wood	400-350, 310-210 BC<br>1000-800 BC	1970:32	Alder keg *K1 with alder cord	3, 10-11
			2165±29; BRAMS-1102.3.1 ($C_{16:0}$ FA)	360-150, 140-110 BC					
			2124±29; BRAMS-1102.4.1 ($C_{16:0}$ FA)	350-320, 210-50 BC					
			2199±37; BRAMS-1102.3.2 ($C_{18:0}$ FA)	380-170 BC					
			2211±35; BRAMS-11-2.4.2 ($C_{18:0}$ FA)	380-190 BC					
IB19	Teernakill Bog (Teernakill North or South)	Galway	509±15; BRAMS-1103.1	AD 1410-1440	840±50; GrA-6015; wood	AD 1040-1100, 1110-1280	1925:14	Plunge churn *PC1	3, 9
			536±37; BRAMS-1103.2.1 ($C_{16:0}$ FA)	AD 1310-1360, 1380-1450					
			499±32; BRAMS-1103.3.1 ($C_{16:0}$ FA)	AD 1320-1340, 1390-1450					
			486±38; BRAMS-1103.2.2 ($C_{18:0}$ FA)	AD 1320-1350, 1390-1470					
			476±29; BRAMS-1103.3.2 ($C_{18:0}$ FA)	AD 1410-1460					
IB20	Ards Beg	Donegal	1198±25; BRAMS-1104.1	AD 720-740, 760-900			1987:112	Tub *T	7
IB21a	Ballyguin	Mayo	899±27; BRAMS-1155.1	AD 1040-1210			1943:314-5	Two bladders in wooden container	3
IB21b			912±27; BRAMS-1155.2	AD 1030-1190					
IB22tp	Derrycoogh	Tipperary	1827±27; BRAMS-1156.1	AD 90-100, 120-260	1850±40; GrN-28754; butter	AD 60-260	1991:13	Bark wrappings	3-5
IB22bm			1829±27; BRAMS-1156.2	AD 90-100, 120-250					
IB23	Derryloughan	Mayo	1177±25; BRAMS-1105.1	AD 770-900, 920-950			M1948:4	Wooden	
IB24	Killinagh	Kildare	2173±26; BRAMS-1106.1	360-160 BC			1929:1298	Wooden	
IB25	Glennacowan	Limerick	1813±26; BRAMS-1107.1	AD 120-260, 300-320			1943:54		
IB26	Ardanew	Meath	1865±26; BRAMS-1108.1	AD 70-230			1930:195	Keg *K2	7
IB27	Rosdoagh	Mayo	448±25; BRAMS-1109.1	AD 1420-1470			1968:440A	Hazel stave tub	3, 7, 12
IB28	------	Leitrim	887±25; BRAMS-1110.1	AD 1040-1100, 1110-1220			2018:29-30	Bark	
IB29	Gowlaune	Mayo	357±25; BRAMS-1111.1	AD 1450-1530, 1550-1640			2018:27-28	Bark	
IB30	Cloncoose	Sligo	2211±26; BRAMS-1112.1	370-200 BC			2007:38	Bark	
IB31	Derragh	Mayo	959±25; BRAMS-1113.1	AD 1020-1160			2007:39		
IB32	Knockmoyle	Mayo	1399±26; BRAMS-1114.1	AD 600-670			1986:39	Keg *K4	3

Code	Townland	County	^{14}C age BP and associated lab code (this study)	Calibrated date range (2σ)	Associated ^{14}C determination (years BP)	Calibrated date range (2σ)	NMI accession no.	Container * Earwood 1997 type	Refs
	Inchimacteige	Kerry			2090±50; GrN-28742	360-300 BC, 210 BC-AD 30	1941:1118	'Firkin'	4, 5
	Lonart	Kerry			1265±35; GrN-28743	AD 660-780, 790-870	M1948:1	Wooden	4
	Goolamore	Mayo			615±30; GrN-28745	AD 1290-1410	1955:45.1-.2	Mether	4, 5
	Enagh	Cavan			925±30; GrN-28746	AD 1020-1190	1959:747	Bladder	4, 5
	Rathbaun	Sligo			1980±50; GrN-28747	120 BC-AD 130	1967:144	Bladder	4, 5
	Crovehy	Donegal			265±30; GrN-28748	AD 1510-1600, 1610-1670, 1780-1800	1967:212.1-.2	Animal skin?	4, 5
	Canburrin	Kerry			970±30; GrN-28749	AD 1010-1160	1971:1006	Cylindrical bark envelope	4
	Annaghbeg	Kerry			2150±50; GrN-28750	360-50 BC	1975:235	Tub	4
	Corragarrow	Longford			670±25; GrN-28751	AD 1270-1320, 1350-1390	1984:152	Wooden keg	4, 5
	Killoran	Tipperary			1840±35; GrN-28753	AD 70-250	1990:119	Wicker	4, 5
	Inchiboy	Kerry			1205±30; GrN-28755	AD 710-750, 760-900, 930-940	1995:97	Bark	4
	------	------			1970±50; GrN-28757	110 BC-AD 140			4, 5
	Clonava	Westmeath			3061±33; UBA-9610	1420-1230 BC	2016:223		
	Drinagh	Offaly			2981±22; UBA-9618	1270-1120 BC	1977:2176	Bark and withies?	
	Morvern	Argyle			1802±35; UB-3185	AD 120-330	NMS ME166	Alder keg	3, 4, 13
	Kyleakin	Skye			1730±35; UB-3186	AD 230-400	NMS ME167	Alder keg	4, 13-14
	High Borve	Lewis			949±20; UB-3737	AD 1025-1155		Two balls wrapped in intestine	3-4, 15

References for Supplementary Table S2

1. Bronk Ramsey C (2017) Methods for Summarizing Radiocarbon Datasets. *Radiocarbon* 59(2):1809-1833.
2. Reimer PJ, Bard E, Bayliss A, Beck JW, Blackwell PG, Bronk Ramsey C, Grootes PM, Guilderson TP, Haflidason H, Hajdas I, Hatté C, Heaton TJ, Hoffmann DL, Hogg AG, Hughen KA, Kaiser KF, Kromer B, Manning SW, Niu M, Reimer RW, Richards DA, Scott EM, Southon JR, Staff RA, Turney CSM, van der Plicht J (2013) IntCal13 and Marine13 Radiocarbon Age Calibration Curves 0-50,000 Years cal BP. *Radiocarbon* 55(4): 1869-1887.
3. Earwood C (1997) Bog Butter: A Two Thousand Year History. *J Irish Archaeol* 8:25-42.
4. Downey L, Synnott C, Kelly EP, Stanton C (2006) Bog butter: dating profile and location. *Archaeol Ireland* 20(1):32-34.
5. Cronin T, Downey L, Synnott C, McSweeney P, Kelly EP, Cahill M, Ross RP, Stanton C (2007) Composition of ancient Irish bog butter. *Int Dairy J* 17:1011-1020.
6. Lucas AT (1964) Archaeological acquisitions in the year 1962. *J Roy Soc Antiq Ireland* 94(2):85-104.
7. Earwood C (1993) *Domestic wooden artefacts in Britain and Ireland from Neolithic to Viking times* (University of Exeter Press, Exeter)
8. Lucas AT (1968) Archaeological acquisitions in the year 1965. *J Roy Soc Antiq Ireland* 98:93-159.
9. Raftery J (1942) A bog butter vessel from near Tuam, Co. Galway. *J Galway Archaeol Hist Soc* 20:31-38.
10. Lucas AT (1973) Archaeological acquisitions in the year 1970. *J Roy Soc Antiq Ireland* 103:177-213.
11. Raftery J (1976) Bog butter find. *J Kildare Archaeol Soc* 15:17-18.

12. Lucas AT (1971) Archaeological acquisitions in the year 1968. *J Roy Soc Antiq Ireland* 101:184-244.
13. Berstan R, Dudd SN, Copley MS, Morgan ED, Quye A, Evershed RP (2004) Characterisation of bog butter using a combination of molecular and isotopic techniques. *Analyst* 129:270-275.
14. Earwood C (1991) Two early historic bog butter containers. *P Soc Antiq Scotland* 121:231-240.
15. Hunter F (1997) Iron Age hoarding in Scotland and northern England. *Reconstructing Iron Age Societies*, eds Gwilt A, Haselgrove C (Oxbow, Oxford), pp 108-27
